# Detecting the stable point of therapeutic effect of chronic myeloid leukemia based on dynamic network biomarkers

**DOI:** 10.1186/s12859-019-2738-0

**Published:** 2019-05-01

**Authors:** Junhua Xu, Min Wu, Shanshan Zhu, Jinzhi Lei, Jie Gao

**Affiliations:** 1 School of Science,Jiangnan University, Wuxi, 214122 China; 2 Zhou Pei-Yuan Center for Applied Mathematics, Tsinghua University, Beijing, 100084 China

**Keywords:** Chronic myeloid leukemia (CML), Dynamic network biomarkers (DNB), Differentially expressed genes (DEGs), Therapeutic effect index (TEI), Pre-stable state, Treatment time

## Abstract

**Background:**

Most researches of chronic myeloid leukemia (CML) are currently focused on the treatment methods, while there are relatively few researches on the progress of patients’ condition after drug treatment. Traditional biomarkers of disease can only distinguish normal state from disease state, and cannot recognize the pre-stable state after drug treatment.

**Results:**

A therapeutic effect recognition strategy based on dynamic network biomarkers (DNB) is provided for CML patients’ gene expression data. With the DNB criteria, the DNB with 250 genes is selected and the therapeutic effect index (*TEI*) is constructed for the detection of individual disease. The pre-stable state before the disease condition becomes stable is 1 month. Through functional analysis for the DNB, some genes are confirmed as key genes to affect the progress of CML patients’ condition.

**Conclusions:**

The results provide a certain theoretical direction and theoretical basis for medical personnel in the treatment of CML patients, and find new therapeutic targets in the future. The biomarkers of CML can help patients to be treated promptly and minimize drug resistance, treatment failure and relapse, which reduce the mortality of CML significantly.

**Electronic supplementary material:**

The online version of this article (10.1186/s12859-019-2738-0) contains supplementary material, which is available to authorized users.

## Introduction

Chronic myeloid leukemia (CML) is a clonal myeloproliferative disorder of a pluripotent stem cell. It is mainly caused by the disorder of differentiation and maturation of hematopoietic stem cells. The annual incidence rate is about 1.3 per 100,000, and it is slightly more common in males than in females. The main hallmark is the presence of the Philadelphia chromosome, which is resulted from the balanced translocation of chromosome t(9;22) (q34; q11) [1]. At present, the use of ABL kinase inhibitors (e.g. imatinib) for the treatment of CML can inhibit the activity of BCR-ABL kinase effectively, inhibit the malignant proliferation of leukemia cells, and extend the survival time of patients significantly. During the treatment, there will be a stable point in CML drug response [2]. The condition of patients gradually eases before it comes, and stabilizes after it comes. It’s difficult to find the stable point only through clinical medicine. Therefore, it’s urgent to discover and validate stable points through bioinformatics for CML drug therapy.

Increasing evidences suggest that many mathematical models can contribute to elucidating mechanisms and providing quantitative predictions for cancer research [3], and the combination of model and clinical information has provided useful suggestions for treatment [4]. Sasaki K et al. used the robust linear regression model to define the best fit average molecular response, where the average molecular levels were defined. Predicting the highest probability of reaching optimal values proposed by the model to decide whether to continue treatment [5]. In addition, traditional biomarkers cannot distinguish the state of critical point before the disease worsens. Based on this situation, Chen LN et al. [6] proposed a theory of dynamic network biomarkers (DNB) to analyze the dynamic signals of DNB when the system was in the critical point state, and put forward three universal properties of DNB [7, 8]. Markus AD et al. showed that the critical point will enter the disease state quickly under certain triggering factors, so the critical point was treated as an early warning signal for complex diseases [9]. Lesterhuis WJ et al. found that the use of dynamic network biomarkers can identify critical points in the state of the system by comparing dynamic biomarkers with static biomarkers of complex diseases [10]. Combined with the advantages of high-throughput sampling of gene expression data, many discussions have shown that DNB is promising candidate biomarker for clinical trials and clinical detection of complex diseases [11].

Based on the advanced high-throughput technology, gene or protein expression data with dynamic measurements can be obtained. In order to detect the therapeutic effect of CML medications from a small amount of high-throughput data, a therapeutic effect recognition strategy is provided based on DNB for CML patients’ gene expression data. In the study, the datasets divided into the treatment group and the control group are used to select differentially expressed genes (DEGs) by *t*-test. DEGs are clustered into 60 categories by hierarchical clustering. Then, according to the three criteria for the identification of DNB proposed by Chen, a group of 250 genes is selected as DNB. Therefore, the therapeutic effect index (TEI) is constructed to observe the dynamic change, and it can be used to predict and determine when it is in pre-stable state. Finally, functional enrichment analysis is performed on the DNB, and the role of the DNB in CML is studied by KEGG enrichment analysis and literature mining.

## Materials and methods

### Datasets

**Fig. 1:**
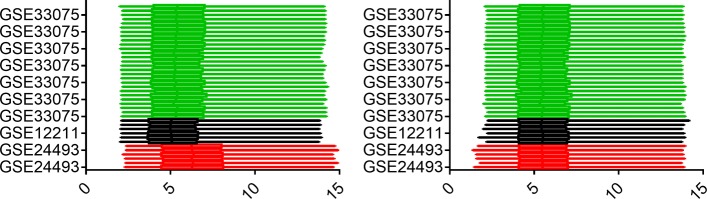
The box plots of data expression. The combined dataset is visually displayed by the gene box plot. On the left side, the three datasets are merged without any transformation. On the right side, the three datasets are merged with the COMBAT method. After removing batch effects, the distribution of genes is more similar than before

Three datasets, including GSE33075, GSE12211, and GSE24493 from the National Center for Biotechnology Information’s Gene Expression Omnibus (GEO) database are used to analyze treatment time. Initially, datasets in CEL files are standardized by Robust Multichip Averaging (RMA) implemented in the affy package, and return the log2 conversion intensity [12], and the probe sets are mapped to unique gene symbols by the averaging method. This study doesn’t consider probe sets without corresponding gene symbols. Due to limited experimental data, multiple GEO data are combined to obtain 39 chips. The information of dataset is shown in Table 1. In the study, samples of CML diagnosed are defined as control groups. 8927 genes can be obtained from the same gene of each GEO dataset. The COMBAT method is used to adjust the batch effect [13]. The experiment Information of dataset is shown in Table 2. Figure 1 shows the distribution of box plots before and after removing batch effects.

**Table 1:** The information of dataset

Dataset	Probe	Gene	Diagnosis	Treatment for 16 h	Treatment for 7 days	Treatment for 1 month	Normal
GSE33075	45782	23507	9	-	-	9	9
GSE12211	21225	13506	-	-	6	-	-
GSE24493	45782	23507	3	3	-	-	-

**Table 2:** The experiment information of dataset

Dataset	Platform	Imatinib used in the experiment
GSE33075	GPL570	400 mg imatinib mesylate (IM)/day
GSE12211	GPL571	400mg Glivec/day
GSE24493	GPL570	10 *μ*M STI571 (Imatinib) for 16 h

Note: STI571’s generic name is imatinib mesylate and its trade name is Glivec

**Fig. 2:**
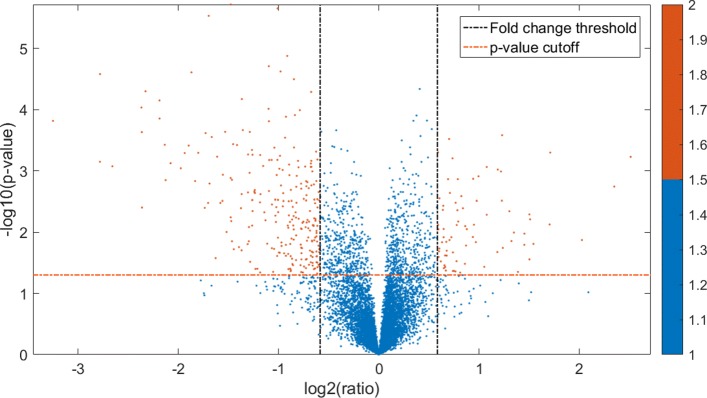
The volcano plot of DEGs

The student’s *t*-test applied in the selection of DEGs is used to assess the significance of DEGs between the control group and the treatment group. The *p*-value calculated by *t*-test is used for the subsequent filtering analysis with multiple testing corrections directly. Set the *p*-value of 0.05 and the fold change of 1.5. The volcano plot is shown in Fig. 2.

### Identify pre-stable state based on DNB

We assume the reference sample data is *C*(*t*), where the n-dimensional vector represents the observed value or molecular concentration (e.g. gene expression or protein expression) at time *t* (*t*=0, 1,...), e.g. minutes, hours or days. Therefore, the Pearson correlation coefficient (*PCC*) [14] between the two genes *x*, *y* in the data from reference sample can be calculated as

1 $$\begin{array}{*{20}l} PCC(x,y)=\frac{\sum_{i=1}^{n} (x_{i}-\bar{x})(y_{i}-\bar{y})}{\sqrt{\sum_{i=1}^{n} (x_{i}-\bar{x})^{2}\sum_{i=1}^{n}(y_{i}-\bar{y})^{2}}} \end{array} $$

Where *x*_*i*_ and *y*_*i*_ represent the *i*−*th* sample’gene expressions of gene *x* and gene *y* in the reference sample, respectively. $\bar {x}$ and $\bar {y}$ represent the average gene expression of gene *x* and gene *y* in the reference sample, respectively.

The reference sample data *C*(*t*) can be divided into two groups, the control group *C*_*control*_(*t*) and the treatment group *C*_*treat*_(*t*), as follows

2 $$\begin{array}{*{20}l} C_{control}(t)=(C_{control}^{1}(t),...,C_{control}^{n}(t)) \end{array} $$

3 $$\begin{array}{*{20}l} C_{treat}(t)=(C_{treat}^{1}(t),...,C_{treat}^{n}(t)) \end{array} $$

**Fig. 3:**
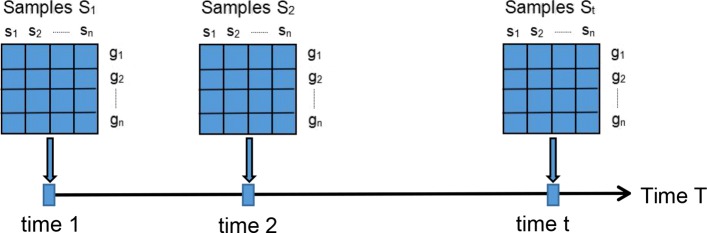
Sampling time and samples for the measured high throughput data

There are *S*_*t*_ samples at time *t* for each gene or protein (see Fig. 3). Due to large differences in the expression values of various genes or proteins, the expression data is standardized as follow

4 $$\begin{array}{*{20}l} \tilde{C}=\frac{C_{treat}-mean(C_{control})}{SD(C_{control})} \end{array} $$

Where $\tilde {C}$ represents the standardized expression data for gene or protein of each sample. *mean*(*C*_*control*_) and *SD*(*C*_*control*_) are the mean and standard deviation in control samples, respectively. Then the standardized matrix is showed

5 $$\begin{array}{*{20}l} \tilde{C}= \left[ \begin{array}{cccc} \tilde{c_{11}}& \tilde{c_{12}}&...&\tilde{c_{1t}}\\ \tilde{c_{21}}&\tilde{c_{22}}&...&\tilde{c_{2t}}\\...&...&...&...\\ \tilde{c_{n1}}&\tilde{c_{n2}}&...&\tilde{c_{nt}} \end{array} \right] \end{array} $$

where $\tilde {c_{nt}}$ denotes the standardized data of the *n*−*th* reference sample at time *t*.

Potential DNB modules can be detected because of the gene expression for a specific sample. For specific samples, DEGs are clustered by hierarchical clustering analysis. According to the three criteria of DNB identification proposed by Chen [15], the optimal group of genes or proteins is selected as DNB and is labeled as *C*_*DNB*_, the rest groups are labeled as *C*_*other*_. During disease treatment, a key point is defined as pre-stable state, where the change of DNB is relatively stable after treatment, and the state changes sharply before pre-stable state. After identifying the DNB, the *TEI* at each time can be constructed based on the following three criteria:

(i) As the system approaches the pre-stable state, the average coefficient variation (*CV*) of molecules in this DNB group decreases rapidly and then approaches the *CV* value of health.

(ii) The average *PCCs* of molecules in this DNB group decreases rapidly in the absolute value and then approaches the *PCC* value of health.

(iii) The average *PCCs* of molecules between this DNB group and outside of DNB group increases rapidly in the absolute value and then approaches the *OPCC* value of health. Therefore, *TEI* at each time can be constructed as:

6 $$\begin{array}{*{20}l} TEI_{t}=\frac{CV_{t} \times{PCC_{t}}}{OPCC_{t}} \end{array} $$

where

7 $$\begin{array}{*{20}l} CV_{t}=\frac{SD(C_{DNB}(t))}{mean(C_{DNB}(t))} \end{array} $$

8 $$\begin{array}{*{20}l} PCC_{t}=\frac{cov(c_{i_{1}t},c_{i_{2}t})}{\sigma(c_{i_{1}t})\sigma(c_{i_{2}t})} \end{array} $$

9 $$\begin{array}{*{20}l} OPCC_{t}=\frac{cov(c_{it},c_{jt})}{\sigma(c_{it})\sigma(c_{jt})} \end{array} $$

(*i*=1, 2,..., the number of DNB)(*j*=1, 2,..., the number outside of DNB)Where *PCC*_*t*_ is the average *PCC* of the DNB group at time *t* in absolute value. *OPCC*_*t*_ is the average *PCC* between the DNB group and the outside of DNB group at time *t* in absolute value. *CV*_*t*_ is the coefficient of variation of the DNB group at time *t*. According to the characteristics of the treatment, the *TEI* value changes slowly at the beginning of treatment, and decreases rapidly to be the lowest(i.e., reaches the pre-stable state) after treatment for a period of time, then approaches the *TEI* value of health.

## Result

**Fig. 4:**
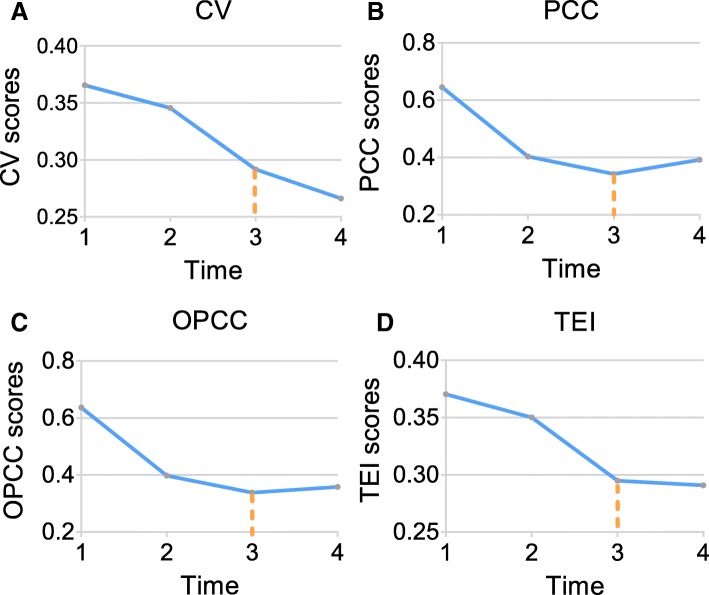
The therapeutic effect index of CML. The abscissa represents time *t*. On the timeline, 1 represents imatinib for 16 h, 2 represents imatinib for 3 days, 3 represents imatinib for 1 month, and 4 represents normal. **a** The average coefficient variation (*CV*) of DNB. **b** The average *PCC* of DNB. **c** The average *PCC* between the DNB group and outside of the DNB group. **d** The *TEI* of DNB

Based on the gene expression of the control group and the treatment group, 321 DEGs are selected by *t*-test and clustered into 60 categories by correlation analysis. A group of 250 genes is identified as the DNB (Additional file 1), where 43 genes relate to CML closely (Additional file 2). In order to clarify the time in the treatment, Fig. 4 shows the changes of four indices in detail. In the progress of imatinib treatment for CML patients, the *CV* value of DNB decreases gradually in Fig. 4a. The *CV* value is the lowest and closest to health value at time 3 (i.e., imatinib treatment for 1 month). The *PCC* value is the lowest at time 3, indicating the correlations of DNB decreases gradually in the process of imatinib treatment and the condition eases gradually in Fig. 4b. Although the change of the *OPCC* is not obvious in Fig. 4c, the *TEI* value is the lowest at time 3 and closest to the *TEI* value of health in Fig. 4d. Therefore, the most significant physiological effect occurs at time 3, indicating that the condition of CML patients is relieved significantly and become normal after imatinib treatment for 1 month.

**Fig. 5:**
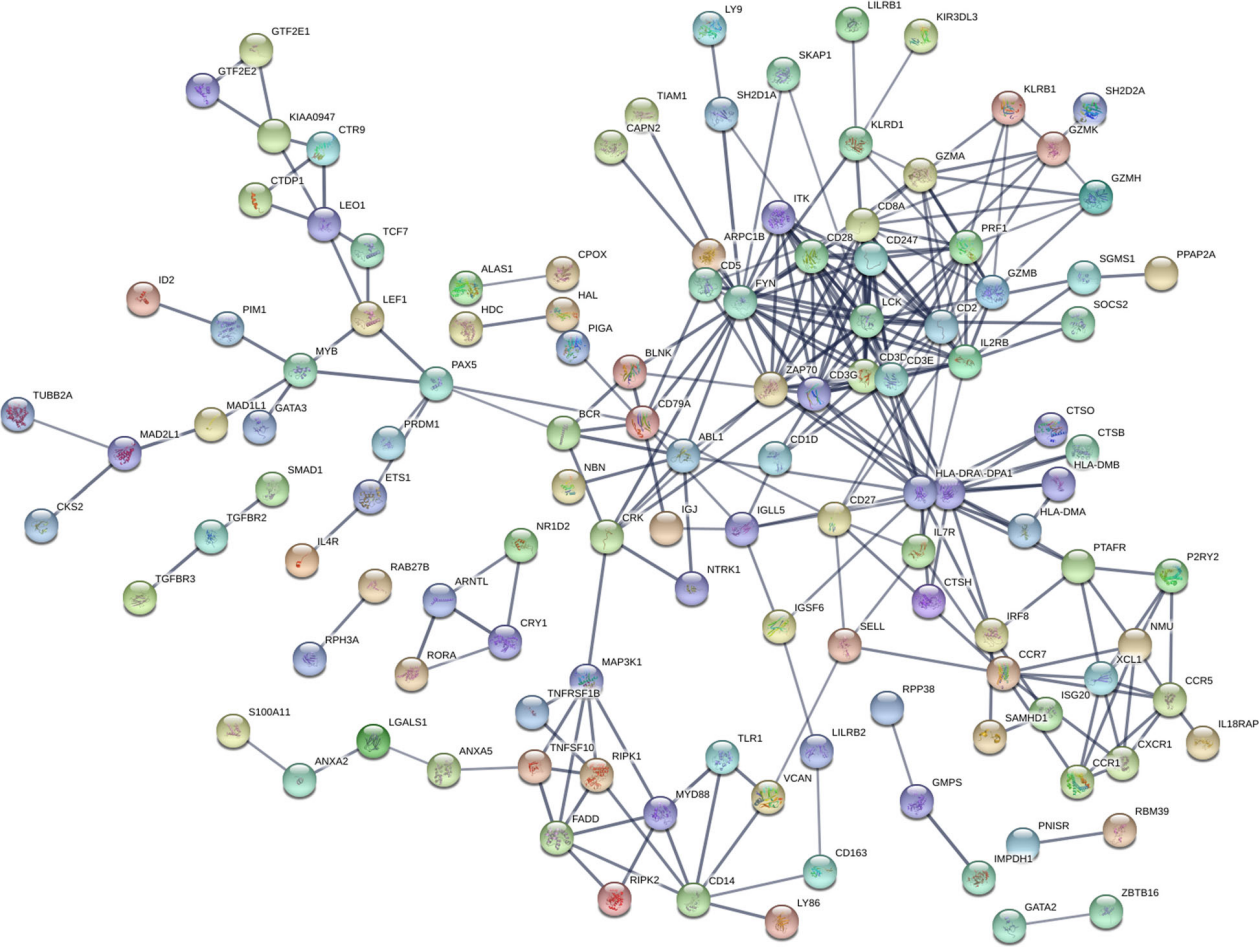
Protein-Protein interaction (PPI) network for part of DNB. PPI network discusses the molecular mechanism of disease from the perspective of the system. A PPI network is set up for 250 DNBs, an interaction score of 0.7 is set, and genes not in the network are deleted. A PPI network of 228 genes is obtained, and it is found that most genes in DNB interact strongly and most of the 42 genes associated with CML have been shown to be most interactive

**Fig. 6:**
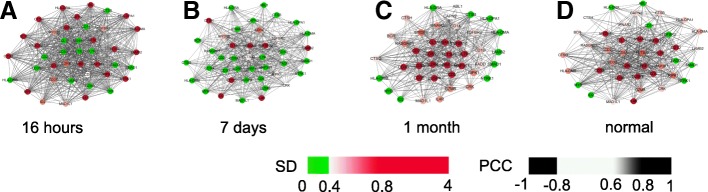
Dynamic changes in DNB (250 genes) subnetwork (43 genes) with 4 sampling points. For CML, we show the dynamic evolution of the network structure of the identified DNB subnetwork with 4 sampling points. (**a**) DNB at 16 h. 43 genes, 631 lines (**b**) DNB at 7 days. 43 genes, 413 lines (**c**) DNB at 1 month (the pre-stable state). 43 genes, 385 lines (**d**) DNB in normal. 43 genes, 457 lines. Each point represents a gene, which is gradually colored according to the standard deviation of the gene. Lines between genes indicate the correlation between genes, calculated by *PCC*, and the lines with weak correlation (|*PCC*|≤0.4) are deleted. From these dynamic evolution charts, it can be clearly seen that the DNB group provides important signals when the system approaches the pre-stable point, the standard deviation of DNB genes becomes smaller and tends to be stable after treatment for 1 month, correlation of DNB genes is gradually weakened and the condition has eased and stabilized. So, a strongly correlated observable subnetwork is also formed in terms of expression variations and network connections

To analyze the DNB dynamics, we discusses the molecular mechanism of disease from the perspective of the system by protein-protein interactions (PPI) in Fig. 5. It can be found that most genes in DNB interact strongly and most of the 43 DNB genes associated with CML have been shown to be most interactive. We also graphically demonstrate the dynamic changes in DNB with 4 sampling points in Fig. 6, which clearly shows the significance of the DNB in terms of expression variations and network structures near the pre-stable point (1 month).

To further analyze the biological function of the DNB, a bioinformatics database DAVID [16] with Gene Ontology (GO) analysis and Kyoto Encyclopedia of Genes and Genomes (KEGG) pathway analysis is provided. GO analysis can be divided into three parts: molecular function, biological process and cellular composition. Some enriched GO functions based on the identified genes in the DNB are listed in Table 3. Some genes have been shown to be associated with CML. For example, on the cellular level, CML is associated with a specific chromosomal abnormality, T (9;22) is reciprocally transposed to form the Philadelphia (PH) chromosome, and the *C*−*ABL* proto-oncogene on chromosome 9 and the *BCR* (breakpoint cluster region) gene on chromosome 22 lead to the PH chromosome. The fusion of *C*−*ABL* and *BCR* is considered to be the main reason of CML. *CRK* is considered as the major tyrosine phosphorylated protein on recognition of CML neutrophils. PI3K is a heterodimer of regulatory and catalytic subunits, and the protein encoded by PIK3R2 is a regulatory component of PI3K. The protein encoded by *TGFBR*2 is a transmembrane protein that has a protein kinase domain, forms a heterodimeric complex with TGF- *β* receptor type-1, and binds TGF- *β*. This receptor/ligand complex phosphorylates proteins, which then enter the nucleus and regulate the transcription of genes related to cell proliferation, cell cycle arrest, wound healing, immunosuppression, and tumorigenesis [17]. The genes mentioned are associated with the pathogenicity of CML and may also regulate and provide an early warning signal for the process of CML treatment.

**Table 3:** Functional enrichment of GO for part of DNB

Enriched items	Genes	*p*-value
Cell surface receptor signaling pathway (GO:0007166)	GCD3G, CD3D, CD8A, CD3E, CCR1, CD247, CXCR1, FADD, IL7R, IL17RA, IFNAR2, LILRB2, TNFSF10, MYD88, CCR5, LILRB3, CD2, KLRD1, CD14, CD27, CD28	5.57E-21
Immune response (GO:0006955)	IL18RAP, AQP9, CD8A, GZMA, CCR1, HLA-DMB, GZMH, IL7R, HLA-DMA, LILRB2, TNFRSF1B, TNFSF10, CCR5, IL4R, IRF8, ZAP70, HLA-DPA1, CD27, PTAFR, HLA-DRA	1.50E-13
T cell costimulation (GO:0031295)	CD3G, TRAC, CD3D, CD3E, LGALS1, CD247, LCK, HLA-DPA1, CD5, HLA-DRA, CD28	4.77E-12
T cell receptor signaling pathway (GO:0050852)	CD3G, TRAC, CD3D, CD3E, GATA3, CD247, LCK, ZAP70, HLA-DPA1, HLA-DRA, PIK3R2, CD28	1.61E-10
Apoptotic process (GO:0006915)	PRF1, GZMA, LGALS1, LY86, TGFBR2, FADD, GZMB, ZBTB16, GZMH, TNFSF10, MYD88, RIPK1, MAP3K1, CD2, CTSH, CD14	1.03E-07

Functional enrichment analysis showed that DNB gene is involved in biological processes such as cell surface receptor signaling pathway, immune response, cell adhesion and apoptotic process. The specific immune responses of CML contribute to the control of the disease. For example, the low expression of antigens recognized by *CD*247 leads to impaired immune response [12], and is also associated with T cell co-stimulation and cell surface receptor signaling pathways. TNF receptor family member *CD*27 is expressed on bone marrow CML stem/progenitor cells in the bone marrow of CML patients. *CD*27 signaling promotes the growth of *BCR*/*ABL*^+^ leukemia cells by activating the Wnt pathway. Therefore, adaptive immunity contributes to leukemic progression. Targeting *CD*27 on the leukemia stem cells (LSCs) may represent an attractive therapeutic approach in blocking the Wnt/ *β*-catenin pathway in CML [13]. Changes in *LGALS*1 expression trigger changes in *MDR*1 expression and resistance to cytotoxic drugs, and *MDR*1 shows high efficacy in the treatment of BCR-ABL-positive CML, so *LGALS*1 may be considered as a novel target for combination therapy, used to improve the efficacy of imatinib in the treatment of CML [18]. Also, it is involved in the process of apoptosis. *TGFBR*2 regulates cell proliferation and participates in apoptotic processes.

**Fig. 7:**
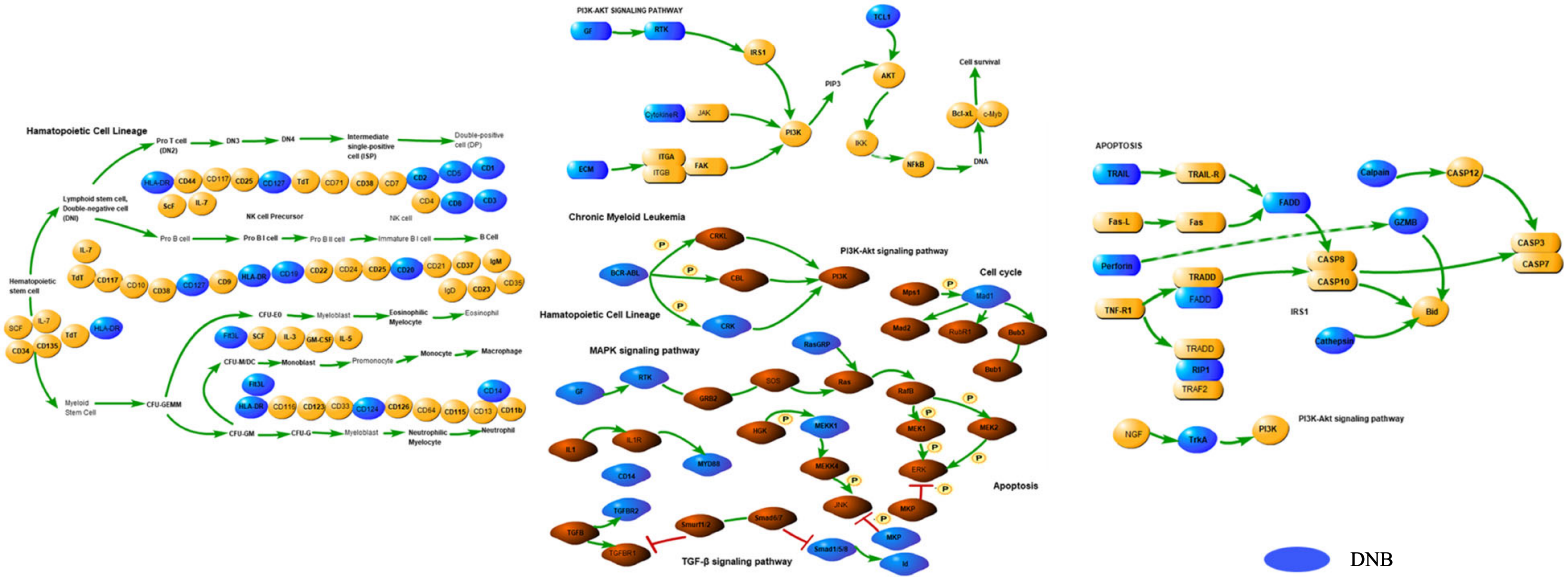
Key biological pathways with DNB genes in CML pathway. By splitting the KEGG pathway map, a portion of the genes associated with DNB are extracted and finally the sub-pathway is obtained, as shown in the above figure. Among them, blue represents DNB, red represents genes in the CML pathway, and yellow represents genes of CML pathway’s pathways. Lines between genes represent various relationships between genes

According to KEGG pathway enrichment analysis, at least 50% of DNB genes are closely related to hematopoietic cell lineage, cytokine-cytokine receptor interaction, apoptosis, chronic myeloid leukemia MAPK signaling pathway, PI3K-Akt signaling pathway and other gene pathways. From the results, *BCR*, *TGFBR*2, *ABL*1, *CRK*, and *PIK*3*R*2 play a decisive role in the pathogenesis of CML from CML pathway in Table 4. Hematopoietic cell lineage, apoptosis, MAPK signaling pathway, and PI3K-Akt signaling pathway play a key role in the process of CML treatment in Fig. 7. The PI3K-Akt signaling pathway is activated by a variety of cellular stimuli or toxic insults and regulates basic cellular functions such as transcription, translation, proliferation, growth, and survival. The mitogen-activated protein kinase (MAPK) cascade is a highly conserved module involved in a variety of cellular functions, including cell proliferation, differentiation, and migration. Apoptosis is a genetically programmed process for the elimination of damaged or redundant cells by activation of caspases (aspartate-specific cysteine proteases).

**Table 4:** Functional enrichment of KEGG pathways for part of DNB

Term	Description	Genes	*p*-value
hsa04640	Hematopoietic cell lineage	CD3G, CD8A, CD3D, CD3E, IL7R, FLT3LG, CD1D, IL4R, MS4A1, CD2, CD5, CD14, HLA-DRA	3.23E-11
hsa04060	Cytokine-cytokine receptor interaction	IFNAR2, TNFRSF1B, IL2RB, TNFSF10, IL18RAP, CCR5, IL4R, CCR1, TGFBR2, CXCR1, IL7R, CD27, IL17RA, FLT3LG	3.87E-07
hsa04210	Apoptosis	TNFSF10, NTRK1, RIPK1, FADD, CAPN2, PIK3R2	3.82E-04
hsa05220	Chronic myeloid leukemia	BCR, TGFBR2, ABL1, CRK, PIK3R2	0.005920
hsa04010	MAPK signaling pathway	DUSP4, RASGRP1, NTRK1, MAP3K1, TGFBR2, CRK, CD14	0.043672
hsa04151	PI3K-Akt signaling pathway	FGFR2, IFNAR2, IL2RB, CD19, IL4R, RXRA, ITGB7, PIK3CD, RAC1, JAK3, IL7R, PIK3R2	0.059021

According to literature mining, it has been found that the chemokine receptor CCR5 plays a role in determining blast malignant properties and localization of extramedullary infiltrations in acute myeloid leukemia (AML) [19]. The cell surface target CD52 is expressed on neural stem cells (NSCs) in a group of patients with AML. *CD*52 is a novel prognostic NSC marker and a potential NSC target in patients with AML and may have clinical significance [20]. *GATA*3 is a sensitive and specific marker for diagnosing acute leukemia with T-cell differentiation and may be a useful complement to the panel of immunophenotypic markers for the diagnostic evaluation of acute leukemia [21]. In addition, genes such as *CEBPD*, *FUT*4, *LILRB*1 and *MVP* play a role in the cure, the treatment, and clinical drug resistance of AML [22], providing theoretical directions for the treatment of CML and finding new therapeutic targets in future.

## Discussion

At present, most researches of CML are focused on the treatment, while a few on the progression of patients after drug treatment. Traditional biomarkers of disease can only distinguish normal state from disease state, and cannot recognize pre-stable state after drug treatment. CML patients are often resistant to conventional chemotherapeutic agents and tyrosine kinase inhibitors. Therefore, the key of the treatment is to control the progression of disease treatment. In order to detect the therapeutic effects of imatinib from a small amount of high-throughput data, a therapeutic effect recognition strategy based on DNB is provided for CML patients’ gene expression data. In the study, the student’s *t*-test applied in the selection of DEGs is used to assess the significance of DEGs between the control group and the treatment group. DEGs are clustered into 60 categories by hierarchical clustering, and a group of 250 genes satisfies the three criteria of DNB. Besides, the values of *CV*, *PCC*, and *OPCC* are calculated to construct *TEI* which is used to detect pre-stable state of CML. *TEI* in treatment progression shows 1 month is the best time for curative effect. In pre-stable state, the *OPCC* is not obvious. The other three indices are significantly related to the theory. After treatment for 1 month, the *CV* of the DNB gene becomes smaller and closer to the *CV* value at the time of health. The correlation between genes is gradually weakened, the condition is relieved and tends to be stable.

Among the 250 genes of DNB, 43 genes have been shown in pathogenesis maps of CML, and *BCR*, *TGFBR*2, *ABL*1, *CRK*, and *PIK*3*R*2 may be the key genes leading to the progression of CML, and the remaining genes have also been found in other types of leukemia like AML. It provides a certain theoretical direction to search for target genes in the future. In clinical medicine, imatinib treatment of CML is difficult to achieve recovery. Most patients adhere to medication after the condition is relieved, so that the patients can survive for a long time. Only a small number of patients can be cured and discontinued.

## Conclusions

The results of this study intend to provide a certain theoretical direction and theoretical basis for medical personnel in the treatment of CML patients, and find new therapeutic targets in the future. The biomarkers of CML can help patients to be treated promptly and minimize drug resistance, treatment failure and relapse, which reduce the mortality of CML significantly. Due to the limited data, there are a few sampling points for collection and it is impossible to predict the pre-stable state fully. In the future we will focus on this important topic and continue to refine the algorithm in later research.

## Additional file

Additional file 1 DNB genes of CML. Based on the gene expression of the control group and the treatment group, 321 DEGs are selected by *t*-test and clustered into 60 categories by correlation analysis. A group of 250 genes is identified as DNB. Supporting Information includes all DNB genes, where 215 genes are down-regulated and 35 genes are up-regulated. (PDF 62 kb)

Additional file 2 Key genes in CML pathway. Among the DNB genes, there are 43 genes related to CML closely. Supporting Information includes the key genes and the pathway each gene belongs to. (PDF 73 kb)

## Abbreviations

CML: Chronic myeloid leukemia
AML: Acute myeloid leukemia
DNB: Dynamic network biomarkers
TEI: The therapeutic effect index
DEGs: The differentially expressed genes
GEO: Gene expression omnibus
RMA: Robust multichip averaging
CV: The average coefficient variation
PCC: The Pearson correlation coefficient
SD: Standard deviation
OPCC: The average PCC between the DNB members and other genes
GO: Gene ontology
KEGG: Kyoto encyclopedia of genes and genomes
PH: Philadelphia
NSCs: Neural stem cells
PPI: Protein-protein interaction

## References

[1] Akram AM, Iqbal Z, Akhtar T, Khalid AM, Sabar MF, Qazi MH, Aziz Z, Sajid N, Aleem A, Rasool M (2017). Presence of novel compound bcr-abl mutations in late chronic and advanced phase imatinib sensitive cml patients indicates their possible role in cml progression. Cancer Biol Ther.

[2] Gao J, Zhang L, Jin PX (2014). Influenza pandemic early warning research on ha/na protein sequences. Curr Bioinforma.

[3] Altrock PM, Liu LL, Michor F (2015). The mathematics of cancer: integrating quantitative models. Nat Rev Cancer.

[4] Roeder I, Horn M, Glauche I, Hochhaus A, Mueller MC, Loeffler M (2006). Dynamic modeling of imatinib-treated chronic myeloid leukemia: functional in sights and clinical implications. Nat Med.

[5] Sasaki K, Kantarjian H, O’rien S, Ravandi F, Konopleva M, Borthakur G, Garcia-Manero G, Wierda W, Daver N, Ferrajoli A (2017). Prediction for sustained deep molecular response of bcr-abl1 levels in patients with chronic myeloid leukemia in chronic phase. Cancer.

[6] Chen LN, Liu R, Liu ZP, Li M, Aihara K (2012). Detecting early-warning signals for sudden deterioration of complex diseases by dynamical network biomarkers. Sci Rep.

[7] Liu R, Yu X, Liu X, Xu D, Aihara K, Chen LN (2014). Identifying critical transitions of complex diseases based on a single sample. Bioinformatics.

[8] Liu X, Chang X, Liu R, Yu X, Chen LN, Aihara K (2017). Quantifying critical states of complex diseases using single-sample dynamic network biomarkers. PLoS Comput Biol.

[9] Vafaee F (2016). Using multi-objective optimization to identify dynamical network biomarkers as early-warning signals of complex diseases. Sci Rep.

[10] Lesterhuis WJ, Bosco A, Millward MJ, Small M, Nowak AK, Lake RA (2017). Dynamic versus static biomarkers in cancer immune checkpoint blockade: unravelling complexity. Nat Rev Drug Discov.

[11] Liu X, Wang Y, Ji H, Aihara K, Chen LN (2016). Personalized characterization of diseases using sample-specific networks. Nucleic Acids Res.

[12] Yang LJ, Chen SH, Wang L, Chen S, Yu Z, Lu YH, Li YQ (2010). Change of expression pattern of cd3 genes in peripheral blood t-cells from cml patients. J Exp Hematol.

[13] Schürch C, Riether C, Matter MS, Tzankov A, Ochsenbein AF (2012). Cd27 signaling on chronic myeloid leukemia stem cells activates wnt target genes and promotes disease progression. J Clin Investig.

[14] Lei XJ, Fang M, Wu FX, Chen LN (2018). Improved flower pollination algorithm for identifying essential proteins. BMC Syst Biol.

[15] Yu X, Zhang J, Sun S, Zhou X, Zeng T, Chen LN (2017). Individual-specific edge-network analysis for disease prediction. Nucleic Acids Res.

[16] Sherman BT, Huang DW, Tan Q, Guo YJ, Bour S, Liu D, Stephens R, Baseler MW, Lane HC, Lempicki RA (2007). David knowledgebase: a gene-centered database integrating heterogeneous gene annotation resources to facilitate high-throughput gene functional analysis. BMC Bioinformatics.

[17] Osaki M, Oshimura M, Ito H (2004). Pi3k-akt pathway: its functions and alterations in human cancer. Apoptosis.

[18] Luo W, Song L, Chen XL, Zeng XF, Wu JZ, Zhu CR, Huang T, Tan XP, Lin XM, Yang Q (2016). Identification of galectin-1 as a novel mediator for chemoresistance in chronic myeloid leukemia cells. Oncotarget.

[19] Mirandola L, Chiriva-Internati M, Montagna D, Locatelli F, Zecca M, Ranzani M, Basile A, Locati M, Cobos E, Kast WM (2012). Notch1 regulates chemotaxis and proliferation by controlling the cc-chemokine receptors 5 and 9 in t cell acute lymphoblastic leukemia. J Pathol.

[20] Blatt K, Herrmann H, Hoermann G, Willmann M, Cerny-Reiterer S, Sadovnik I, Herndlhofer S, Streubel B, Rabitsch W, Sperr WR (2014). Identification of campath-1 (cd52) as novel drug target in neoplastic stem cells in 5q-patients with mds and aml. Clin Cancer Res Off J Am Assoc Cancer Res.

[21] Dorfman DM, Morgan EA, Pelton A, Unitt C (2017). T-cell transcription factor gata-3 is an immunophenotypic marker of acute leukemia with t-cell differentiation. Hum Pathol.

[22] Tregnago C, Manara E, Zampini M, Bisio V, Borga C, Bresolin S, Aveic S, Germano G, Basso G, Pigazzi M (2016). Creb engages c/ebp *δ* to initiate leukemogenesis. Leukemia.

