# Supplementary material for: Detecting the stable point of therapeutic effect of chronic myeloid leukemia based on dynamic network biomarkers
Source: BMC Bioinformatics. 2019 May 1;20(Suppl 7):202. doi: 10.1186/s12859-019-2738-0 (PMC6509869; doi:10.1186/s12859-019-2738-0)
Supplement: Supplementary file 1 — DNB genes of CML. Based on the gene expression of the control group and the treatment group, 321 DEGs are selected by t-test and clustered into 60 categories by correlation analysis. A group of 250 genes is identified as DNB. Supporting Information includes all DNB genes, where 215 genes are down-regulated and 35 genes are up-regulated. (PDF 62 kb) [file 12859_2019_2738_MOESM1_ESM.pdf]

DNB genes	up/down
ABL1	up
ACYP1	up
AIM1	down
AKR1B1	down
ALAS1	up
ALG11 /// UTP14C	down
ALPL	down
ANXA2	down
ANXA5	down
AQP9	down
ARNTL	down
ARPC1B	down
ASF1A	down
ASGR2	down
BCL3 /// MIR8085	down
BCR	down
BLNK	down
BTN3A3	down
C6orf120	down
CAMK4	down
CAPN2	down
CCDC69	down
CCR1	down
CCR5	down
CCR7	down
CD14	down
CD160	down
CD163	down
CD1D	down
CD2	down
CD247	down
CD27	down
CD28	down
CD3D	down
CD3E	down
CD3G	down
CD5	down
CD52	down
CD79A	down

CD8A	down
CDR2 /// LOC101060399	down
CEBPD	down
CKS2	up
CLC	up
CPOX	down
CREB5 /// LOC401317	down
CRK	down
CRTAM	down
CTDP1	down
CTR9	down
CTSB	down
CTSH	down
CTSO	down
CXCR1	down
DMXL2	up
DUSP4	down
ELK3	down
ENC1	down
ETS1	down
F5	down
FADD	down
FADS1 /// MIR1908	up
FAIM3	down
FAM216A	up
FANCL	up
FCGR1A /// FCGR1B /// FCGR1C	down
FGF13	down
FLT3LG	down
FUT4	up
FYN	down
GATA2	up
GATA3	down
GCA	down
GCLM	down
GOLGA8A	up
GPR171	down
GPRASP1	down
GTF2E1	down
GZMA	down

GZMB	down
GZMH	down
GZMK	down
GZMM	down
HAL	up
HDC	up
HDHD1	down
HLA-DMA	down
HLA-DMB	down
HLA-DPA1	down
HLA-DQB1 /// LOC101060835	down
HLA-DRA	down
ICE1	down
ICOS	down
ID2	down
ID2 /// ID2B	down
IFI16	down
IFI30 /// PIK3R2	down
IFNAR2	down
IGH /// IGHA1 /// IGHA2	down
IGHA1 /// IGHG1 /// IGHM /// IGHV3-23 /// IGHV4-31	down
IGHD	down
IGHM	down
IGJ	down
IGK /// IGKC	down
IGKC	down
IGLC1	down
IGLC1 /// IGLV3-25 /// IGLV3-25	down
IGLV1-44	down
IGSF6	down
IL10RB	down
IL17RA	down
IL18RAP	down
IL2RB	down
IL32	down
IL4R	down
IL7R	down
IMPDH1	down
IRF8	down

ISG20	down
ITK	down
KCNJ15	down
KCTD12	down
KDM7A	down
KIAA0040	down
KIAA0232	down
KIAA0355	down
KIAA0513	down
KIAA0930	down
KIR3DL1 /// KIR3DL2	down
KIR3DL3	down
KLF11	down
KLRB1	down
KLRC3	down
KLRC4-KLRK1 /// KLRK1	down
KLRD1	down
LAMB2	down
LCK	down
LDLRAD4	down
LEF1	down
LGALS1	down
LILRB1	down
LILRB2	down
LILRB3	down
LOC101928620 /// POU2AF1	down
LSR	down
LY86	down
LY9	down
MAD1L1	down
MAL	down
MAN1A1	down
MAP3K1	down
METTL18	down
MFHAS1	down
MFSD5	down
MRFAP1L1	down
MS4A1	down
MTMR11	down
MVP	down

MYB	down
MYD88	down
NACC2	down
NAIP	down
NAT1	down
NBN	up
NELL2	down
NET1	up
NMU	down
NR1D2	down
NTRK1	up
P2RY2	up
PAX5	down
PGRMC2	down
PIGA	up
PIM1	down
PLCL1	down
PNISR	up
PPAP2A	up
PPP1R16B	down
PPP1R3D	down
PRDM1	down
PRF1	down
PRKRA	up
PSPH	up
PTAFR	down
PTPRN2	down
RAB27B	up
RAB29	down
RASGRP1	down
RBM39	up
REEP5	down
RHD	down
RIPK1	down
RIPK2	up
RNASE6	down
RORA	down
RPH3A	down
RPP38	down
S100A11	down

SAMHD1	down
SECTM1	down
SELL	down
SERINC1	down
SERINC5	down
SETBP1	down
SETD1B	down
SGMS1	down
SGSH	down
SH2D1A	down
SH2D2A	down
SKAP1	down
SLC16A6	down
SLC22A4	down
SLC25A36	up
SLC2A1	down
SLC31A2	up
SLC4A1	up
SLC9A8	down
SMAD1	down
SMPDL3A	down
SOCS2	up
SPOCK2	down
ST3GAL5	down
STT3A	up
TBC1D9	down
TCF7	down
TCL1A	down
TEX30	up
TGFBI	down
TGFBR2	down
TGFBR3	down
TIAM1	down
TLR1	down
TNFRSF1B	down
TNFSF10	down
TNFSF8	down
TOB1	up
TRAC /// TRAJ17 /// TRAV20 /// TRDV2	down

TRBC1	down
TRIB2	down
TUBB2A	down
TYMP	down
VCAN	down
VOPP1	down
XCL1	down
XCL1 /// XCL2	down
ZAP70	down
ZBTB11	up
ZBTB16	up
ZNF200	down
ZNF217	down
