# Supplementary material for: Detecting the stable point of therapeutic effect of chronic myeloid leukemia based on dynamic network biomarkers
Source: BMC Bioinformatics. 2019 May 1;20(Suppl 7):202. doi: 10.1186/s12859-019-2738-0 (PMC6509869; doi:10.1186/s12859-019-2738-0)
Supplement: Supplementary file 2 — Key genes in CML pathway. Among the DNB genes, there are 43 genes related to CML closely. Supporting Information includes the key genes and the pathway each gene belongs to. (PDF 73 kb) [file 12859_2019_2738_MOESM2_ESM.pdf]

Key genes	Pathway	Up/down
ABL1	Chronic myeloid leukemia<br>Cell Cycle	up
BCR	Chronic myeloid leukemia	down
CAPN2	Apoptosis	down
CD14	Hamatopoietic Cell Lineage<br>MAPK signaling pathway	down
CD1D	Hamatopoietic Cell Lineage	down
CD2	Hamatopoietic Cell Lineage	down
CD3D	Hamatopoietic Cell Lineage	down
CD3E	Hamatopoietic Cell Lineage	down
CD3G	Hamatopoietic Cell Lineage	down
CD5	Hamatopoietic Cell Lineage	down
CD8A	Hamatopoietic Cell Lineage	down
CRK	Chronic myeloid leukemia<br>MAPK signaling pathway	down
CTSB	Apoptosis	down
CTSH	Apoptosis	down
CTSO	Apoptosis	down
DUSP4	MAPK signaling pathway	down
FADD	Apoptosis	down
FLT3LG	Hamatopoietic Cell Lineage<br>PI3K-Akt signaling pathway<br>MAPK signaling pathway	down
GZMB	Apoptosis	down
HLA-DMA	Hamatopoietic Cell Lineage	down
HLA-DMB	Hamatopoietic Cell Lineage	down
HLA-DPA1	Hamatopoietic Cell Lineage	down
HLA-DRA	Hamatopoietic Cell Lineage	down
ID2	TGF-β signaling pathway	down
PIK3R2	Chronic myeloid leukemia	down
IFNAR2	PI3K-Akt signaling pathway	down
IL2RB	PI3K-Akt signaling pathway	down
IL4R	Hamatopoietic Cell Lineage<br>PI3K-Akt signaling pathway	down
IL7R	Hamatopoietic Cell Lineage<br>PI3K-Akt signaling pathway	down
LAMB2	PI3K-Akt signaling pathway	down
MAD1L1	Cell Cycle	down
MAP3K1	MAPK signaling pathway	down
MS4A1	Hamatopoietic Cell Lineage	down

MYB	PI3K-Akt signaling pathway	down
MYD88	MAPK signaling pathway	down
NTRK1	Apoptosis<br>PI3K-Akt signaling pathway<br>MAPK signaling pathway	up
PRF1	Apoptosis	down
RASGRP1	MAPK signaling pathway	down
RIPK1	Apoptosis	down
SMAD1	TGF-β signaling pathway	down
TCL1A	Chronic myeloid leukemia<br>PI3K-Akt signaling pathway	down
TGFBR2	TGF-β signaling pathway<br>MAPK signaling pathway	down
TNFSF10	Apoptosis	down
